# Disrupted Brain Structure–Function Coupling and Its Mediating Effects on the Associations Between Cerebral Small Vessel Disease Burden and Cognitive Dysfunction

**DOI:** 10.1111/cns.70604

**Published:** 2025-09-18

**Authors:** Na Wang, Lingfei Guo, Yian Gao, Chaofan Sui, Xinyue Zhang, Yuanyuan Wang, Yajie Fu, Nan Zhang, Yena Che, Hongwei Wen, Changhu Liang

**Affiliations:** ^1^ Key Laboratory of Endocrine Glucose and Lipids Metabolism and Brain Aging, Ministry of Education, Department of Radiology Shandong Provincial Hospital Affiliated to Shandong First Medical University Jinan Shandong China; ^2^ Department of Radiology, China‐Japan Friendship Hospital (Institute of Clinical Medical Sciences) Chinese Academy of Medical Sciences and Peking Union Medical College Beijing China; ^3^ Department of Radiology, Beijing Tongren Hospital Capital Medical University Beijing China; ^4^ Binzhou Medical University Yantai Shandong China; ^5^ Department of Medical Ultrasound West China Hospital of Sichuan University Chengdu Sichuan China; ^6^ Department of Medical Genetics Shandong Provincial Hospital Affiliated to Shandong First Medical University Jinan Shandong China; ^7^ Prenatal Diagnosis Center Shandong Provincial Hospital Affiliated to Shandong First Medical University Jinan Shandong China; ^8^ School of Medical Imaging North Sichuan Medical College Nanchong Sichuan China

**Keywords:** ALFF–GMV coupling, cerebral small vessel disease, mediation analysis, ReHo–GMV coupling, structure–function coupling

## Abstract

**Aims:**

We aimed to specify relationships among cerebral small vessel disease (CSVD) burden, cognitive dysfunction, and brain structure–function coupling changes.

**Methods:**

A total of 108 patients with mild CSVD burden (CSVD‐m), 53 patients with severe CSVD burden (CSVD‐s), and 76 healthy controls (HC) were included in this study. The ratio of regional homogeneity (ReHo) or amplitude of low‐frequency fluctuation (ALFF) to gray matter volume (GMV) was calculated as an indicator of voxel‐wise structure–function coupling.

**Results:**

Significantly decreased or increased ReHo–GMV and ALFF–GMV coupling values in patients with severe CSVD burden were primarily found in several brain regions, and the disrupted structure–function coupling in the right putamen mediated the relationship between CSVD burden and cognitive dysfunction.

**Conclusions:**

Brain structure–function coupling characterized by ReHo–GMV and ALFF–GMV mediated the cognitive dysfunction caused by CSVD and was an innovative and effective brain imaging indicator for exploring the association between CSVD burden and cognitive dysfunction.

## Graphical abstract

This study included 108 patients with mild CSVD burden (CSVD‐m), 53 patients with severe CSVD burden (CSVD‐s), and 76 healthy controls (HC). The ratio of regional homogeneity (ReHo) or amplitude of low‐frequency fluctuation (ALFF) to gray matter volume (GMV) was calculated as an indicator of voxel‐wise structure–function coupling. Mediation analysis indicated that brain structure–function coupling modulated the relationship between CSVD burden and cognitive function.

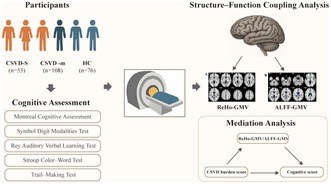

## 1 Introduction

Cerebral small vessel disease (CSVD) manifests as a syndrome with diverse clinical manifestations and neuroimaging features due to structural changes in cerebral blood vessels and the brain parenchyma [1, 2]. The symptoms of CSVD are variable, ranging from asymptomatic imaging markers to the presence of various neuropsychological symptoms, mainly including cognitive dysfunction, depression, stroke, and even dementia [1, 3, 4]. Existing evidence suggests that approximately 20% of strokes are caused by CSVD, which includes 25% ischemic strokes and 45% dementia [1]. The assessment of CSVD severity usually uses the “total burden of small vessel disease [5]” which scores the magnetic resonance imaging (MRI) markers of CSVD. Studies have shown that the total burden of CSVD is associated with disruption of blood–brain barrier integrity and subsequent cognitive impairment, reduced health‐related quality of life after stroke, and poststroke depression [6, 7]. Therefore, it is necessary for researchers to stratify patients by CSVD severity according to the CSVD burden.

Few studies have analyzed alterations in structure–function coupling, although the combination of functional and structural MRI can improve diagnostic accuracy relative to either method alone. In general, the structure–function coupling of the brain is tight, whereas decoupling occurs in neurological diseases [8], which manifests as an increase or decrease in the coupling value. Resting‐state functional MRI (rs–fMRI) parameters, which relies on spontaneous fluctuations in blood oxygen level‐dependent (BOLD) signals [9, 10], coupled with gray matter volume (GMV) are commonly used as structure–function coupling indicators [11]. As voxel‐based BOLD signal metrics, regional homogeneity (ReHo) and the amplitude of low‐frequency fluctuation (ALFF) are considered to represent the local brain characteristics of spatially discrete regions [9]. ReHo reflects the consistency of neuronal activity [12], and ALFF reflects the strength of spontaneous activity of neurons [13]. Kang et al. [14] calculated whole‐brain structure–function coupling via ReHo–GMV and reported the presence of coupling alterations in multiple brain regions in radiation encephalopathy. Zhao et al. [13] calculated whole‐brain structure–function coupling based on the ALFF–GMV and reported decreased coupling in the parahippocampal gyrus and hippocampus, which was also significantly positively related to the severity of cognitive impairment in patients with Alzheimer's disease. These findings suggest the important application of structure–function coupling indicators in brain diseases and their ability to mediate cognitive impairment. Therefore, the exploration of structure–function coupling conditions in CSVD is extremely meaningful and indispensable.

In our recent study, we noted that increased CSVD burden was in connection with reduced ALFF–GMV coupling value in the basal ganglia, increased ALFF–GMV coupling value in the frontotemporal lobe, and more severe cognitive decline [15]. However, the specific relationships among CSVD burden, brain structure–function coupling changes, and cognitive performance remain unclear. A previous study involving mediation analysis [16] revealed a significant mediating effect of network efficiency on age‐related decline in cognitive function, and cerebral amyloid angiopathy altered cognition by causing cortical atrophy, altering cerebrovascular reactivity, and disrupting white matter [17]. As both structural and functional changes in CSVD can affect cognitive function, it is reasonable to ask whether changes in structure–function coupling can also affect cognition. In this study, we aimed to first characterize brain voxel‐wise structure–function coupling on the basis of ALFF/ReHo and GMV metrics and then explore the relationships among CSVD burden, brain structure–function coupling, and cognitive function changes, especially to quantitatively analyze the possible mediating role of disrupted brain structure–function coupling between CSVD burden and cognitive function changes.

## 2 Methods

### 2.1 Participants

Approved by the institutional review board of Shandong Provincial Hospital Affiliated with Shandong First Medical University, 53 patients with severe CSVD burden (CSVD‐s; 19 females; 63.90 ± 6.23 years) and 108 patients with mild CSVD burden (CSVD‐m; 51 females; 61.45 ± 7.79 years) were recruited between December 2018 and January 2022. We also included 76 healthy controls (HC; 42 females; 60.55 ± 9.29 years) matched for age, gender, and education. All participants were right‐handed. Each participant signed an informed consent form voluntarily prior to the start of the study.

Inclusion criteria for CSVD patients included a diagnosis of cerebral microbleeds (CMBs), lacunes of presumed vascular origin, enlarged perivascular space (EPVS), recent small subcortical infarcts, brain atrophy, and white matter hyperintensities (WMHs) of presumed vascular origin, on the basis of current MRI consensus standards [18]. The severity of CSVD was assessed via the “total burden of small vessel disease [5]”, a pragmatic ordinal scale of 0 to 4 based on the first four of the previously mentioned MRI markers of CSVD. One point was awarded for each of the following MRI markers: (1) ≥ 1 lacune; (2) early confluent deep WMH (Fazekas score [19] 2 or 3) or irregular periventricular WMH extending into the deep white matter (Fazekas score 3); (3) moderate to severe (Grades 2–3) EPVS in the basal ganglia; and (4) ≥ 1 CMB. Subjects with scores of 0 to 1 and 2 to 4 were, respectively, included in the CSVD‐m group and CSVD‐s group.

Exclusion criteria were as follows: (1) a history of brain tumors, brain trauma, stroke, or epilepsy; (2) a history of substance or alcohol abuse; (3) a history of major neurologic or psychiatric illness; (4) a history of thrombolysis; (5) acute complications of type 2 diabetes or severe hypertension; and (6) the presence of organ damage (e.g., heart, liver, or kidney).

### 2.2 MRI Acquisition

MRI scans were obtained via a 3.0‐Tesla MR system (Siemens Healthcare, Erlangen, Germany) equipped with a 32‐channel head coil for signal reception. Magnetization‐prepared rapid gradient echo (MPRAGE) sequence was used for acquiring high‐resolution 3D T1‐weighted (T1W) structural images; parameters were as follows: echo time (TE) = 2.4 ms; repetition time (TR) = 7.3 ms; inversion time (TI) = 900 ms; flip angle = 9°; matrix size = 256 × 256; field of view (FOV) = 240 mm × 240 mm; slice thickness = 0.9 mm with no gap and 192 slices. Gradient‐recalled echo echo‐planar imaging (GRE‐EPI) sequence was used for acquiring rs‐BOLD‐fMRI with the following parameters: TR = 1500 ms; TE = 30 ms; matrix size = 64 × 64; FOV = 240 mm × 240 mm; slice thickness = 3 mm; slice gap = 1 mm; 200 volumes; and 50 axial slices.

### 2.3 Cognitive Assessments

Several neuropsychological testing scales were used for the cognitive assessment of all participants. These tests include the Montreal Cognitive Assessment (MoCA) Beijing version (http://www.mocatest.org), a cognitive screening tool with a total score of 30 points and show high sensitivity to mild cognitive impairment [20]; the symbol digit modalities test (SDMT) to assess attention and information processing speed [21]; the Rey auditory verbal learning test (AVLT) to assess memory [22]; the Stroop color‐word test (SCWT) to assess cognitive flexibility and control, and executive function [23]; and the trail‐making test (TMT) to evaluate visual search, information processing speed, motor coordination, and attention [24]. The testing personnel were professionally trained and were blinded to the subject grouping.

### 2.4 Data Preprocessing and Structure–Function Coupling Analysis

In accordance with our recent coupling research [15], the main preprocessing steps for fMRI and T1W images were performed via the Data Processing and Analysis for Brain Imaging toolbox (DPABI v6.0, http://rfmri.org/dpabi). The first 10 volumes were discarded, followed by slice timing and head motion correction. Subjects with head motion exceeding 3 mm/3° or mean framewise displacement (FD) > 0.2 mm [25] were excluded. T1 images were co‐registered, segmented into GM/WM/CSF, and normalized to MNI space using the DARTEL algorithm [26]. Functional images were also normalized, resampled to 3 × 3 × 3 mm^3^, and spatially smoothed with a 4 mm Gaussian kernel. Nuisance covariates (CSF, WM, Friston‐24 motion parameters [27]) were regressed out. Standardized ALFF and ReHo maps were calculated after bandpass filtering (0.01–0.08 Hz [28]), with ReHo computed prior to smoothing. These metrics were used to construct voxel‐wise structure–function coupling maps. The detailed steps were described in Appendix S1 (Data preprocessing).

For each subject, as the structural T1W image had been co‐registered to the functional image, the standardized ReHo/ALFF maps and the modulated GMV maps were also normalized to the same Montreal Neurological Institute (MNI) coordinate system. Then, we generated the voxel‐wise ALFF–GMV and ReHo–GMV coupling metrics by counting the ratio of the ALFF or ReHo value to the GMV value of the voxel at the equal coordinate, respectively.

### 2.5 Statistical Analysis

For demographic and clinical parameters, one‐way analysis of variance (ANOVA) and least significant difference (LSD) post hoc tests were used for assessing differences in age, education, head motion, and cognitive scores among groups, and with the chi‐square test for analyzing the gender ratio.

For the ALFF–GMV and ReHo–GMV coupling maps, one‐way analysis of covariance (ANCOVA) was performed to identify differences among groups after controlling for age, gender, education, and head motion (mean framewise displacement (FD)) as covariates. LSD post hoc tests were subsequently performed, and the corrected *p* values for pairwise group comparisons were calculated. Finally, the post hoc *p* values were converted to *z*‐values, and statistical inferences were made on the *z*‐value maps after Gaussian random field correction via DPABI utilities [29, 30], with voxel‐level and cluster‐level set separately as *p* < 0.001 and *p* < 0.05. For brain clusters with significant intergroup differences, the peak voxel coordinates were reported in the MNI space and then anatomically defined via the automated anatomical labeling atlas [31]. We further extracted the mean ReHo–GMV and ALFF–GMV coupling values from the identified significant clusters for subsequent mediation analysis.

Furthermore, we examined whether brain structure–function coupling could modulate the relationship between CSVD burden and cognitive function using mediation analysis. Briefly, we treated the CSVD burden score as an independent variable; the cognitive score as the dependent variable; the ALFF–GMV or ReHo–GMV coupling metric as the mediating variable; and age, gender, education, and head motion as covariates. Specifically, we employed the simple mediation model (Model 4) in the Hayes' Process macro [32] for SPSS v21.0 software (SPSS Inc., Chicago, IL), incorporating a bootstrapping procedure with 5000 resamples to generate estimates and confidence intervals (CIs). Bootstrapping, a nonparametric resampling strategy [33], aids in estimating and testing indirect effects against zero by creating CIs. In bootstrap‐based mediation analysis, statistical significance is typically determined by whether the confidence interval for the indirect effect excludes zero, rather than by the *p*‐value. An indirect effect was deemed significant if the bootstrapped 95% CIs did not encompass zero [33]. Therefore, *p*‐value correction is generally not required [34, 35, 36].

## 3 Results

### 3.1 Participant Characteristics

The demographic, clinical, and cognitive parameters of all the groups are summarized in Table 1. Compared with the CSVD‐m and HC groups, the CSVD‐s group had significantly lower MoCA, AVLT, and SDMT scores, and higher SCWT and TMT scores. Additionally, the CSVD‐m group had significantly lower SDMT scores and higher SCWT scores than the HC group. There were no significant differences in age, gender, education, or head motion between the groups.

**TABLE 1:** Demographic, clinical, and cognitive parameters for all groups.

Parameters	CSVD‐s	CSVD‐m	HC	*p* (ANOVA/*χ* ^2^)	*p* (post hoc)		
					CSVD‐s vs. HC	CSVD‐s vs. CSVD‐m	CSVD‐m vs. HC
Gender	34 M/19 F	57 M/51 F	34 M/42 F	0.094^a^	—	—	—
Age (years)	63.90 ± 6.23	61.45 ± 7.79	60.55 ± 9.29	0.060^b^	—	—	—
Education (years)	11.26 ± 3.22	12.07 ± 3.14	12.67 ± 3.35	0.053^b^	—	—	—
MoCA	24.47 ± 2.91	25.38 ± 3.57	26.39 ± 3.57	**0.009** ^b^	**0.002**	0.121	0.052
AVLT	54.66 ± 13.5	61.05 ± 12.19	64.6 ± 11.94	**< 0.001** ^b^	**< 0.001**	**0.003**	0.059
SDMT	26.56 ± 11.94	32.16 ± 12.23	40.57 ± 13.53	**< 0.001** ^b^	**< 0.001**	**0.010**	**< 0.001**
SCWT	178.68 ± 59.41	145.47 ± 44.98	131.78 ± 31.08	**< 0.001** ^b^	**< 0.001**	**< 0.001**	**0.045**
TMT (B‐A)	161.8 ± 91.09	125.59 ± 106.34	104.84 ± 80.08	**0.005** ^b^	**0.001**	**0.028**	0.152
FD_Jenkinson	0.13 ± 0.04	0.12 ± 0.04	0.11 ± 0.04	0.135^b^	—	—	—

*Note:* Significance of bold values indicates the statistical significance of *p* values when *p* < 0.05.

Abbreviations: AVLT, sum of Rey auditory verbal learning test (N1‐7); CSVD‐m, mild CSVD burden (score ≤ 1) group; CSVD‐s, severe CSVD burden (score ≥ 2) group; FD_Jenkinson, frame‐wise displacement; HC, healthy controls; MoCA, Montreal Cognitive Assessment; SCWT, sum of Stroop color‐word test (stroop1‐3); SDMT, symbol digit modalities test; TMT, the trail‐making test; TMT (B–A), the difference between TMT‐B and TMT‐A.

^a^ Chi‐square test.

^b^ ANOVA test.

### 3.2 Significantly Altered Structure–Function Coupling in CSVD

**FIGURE 1:**
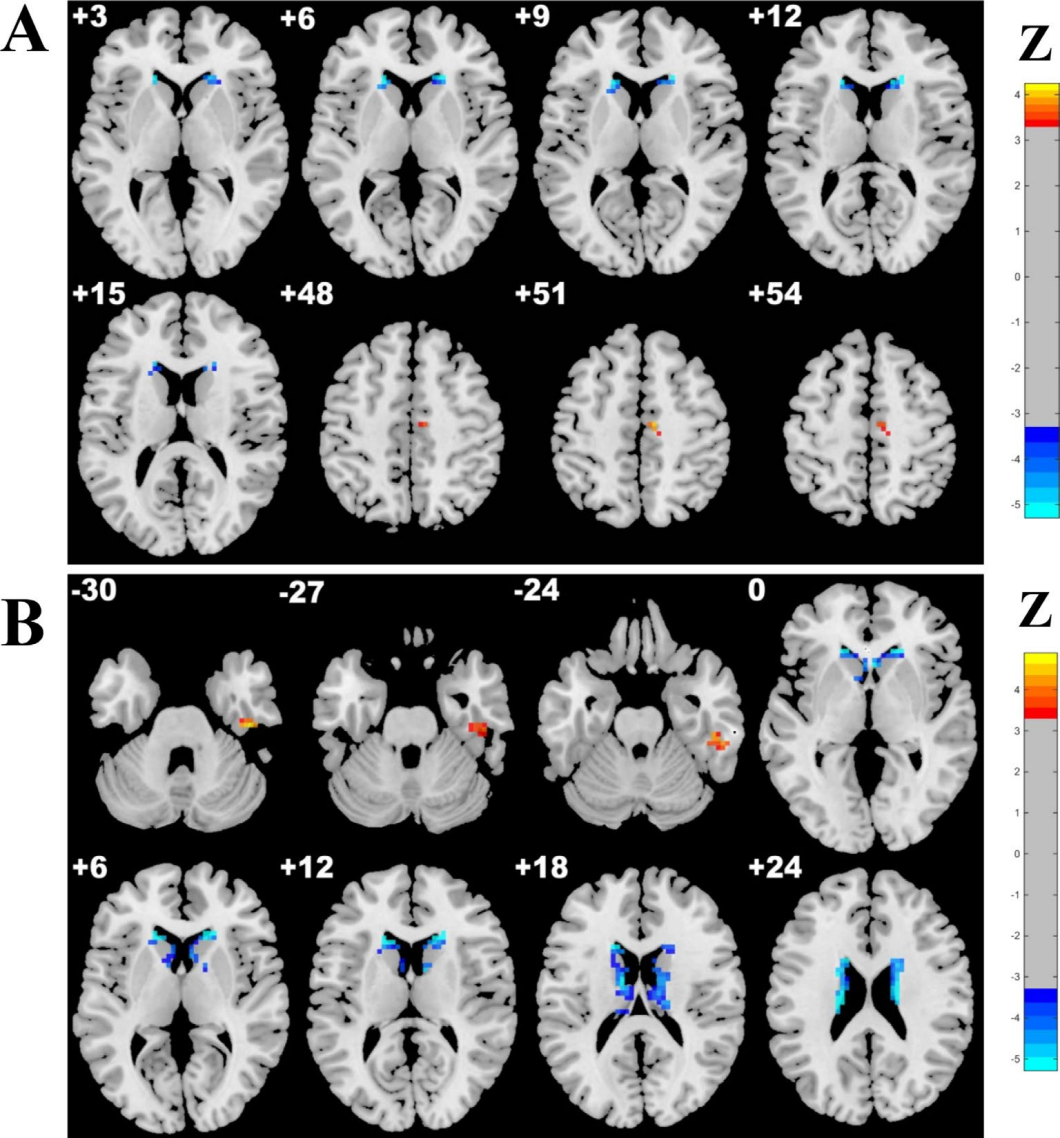
Brain clusters with significantly altered structure–function coupling in the CSVD‐s group relative to the control group. Clusters showing significant differences in (A) ReHo–GMV and (B) ALFF–GMV coupling were identified using ANCOVA followed by LSD post hoc tests, with Gaussian random field (GRF) correction (voxel‐level *p* < 0.001, cluster‐level *p* < 0.05). Only regions with significant changes in the CSVD‐s group compared to controls are shown. As illustrated, increased ALFF–GMV coupling was observed in the right inferior temporal gyrus (ITG), and increased ReHo–GMV coupling in the right supplementary motor area (SMA). In contrast, decreased ALFF–GMV and ReHo–GMV coupling were found in the bilateral caudate nucleus. The red‐yellow and blue‐light blue color bars indicate increased and decreased coupling, respectively, in the CSVD‐s group.

Compared with the HC group, the CSVD‐s group presented significantly decreased ALFF–GMV and ReHo–GMV coupling values in the clusters of the bilateral caudate nucleus (CAU), as well as significantly increased ALFF–GMV coupling in the right inferior temporal gyrus (ITG) and ReHo–GMV coupling in the right supplementary motor area (SMA) (Table 2 and Figure 1).

**TABLE 2:** Brain clusters with significantly altered ReHo‐GMV or ALFF–GMV coupling in CSVD‐s, CSVD‐m, and control groups.

Group	Coupling metrics and condition	Brain regions	Cluster size	*z*‐score of peak voxel	MNI coordinates of peak voxel		
					*x*	*y*	*z*
CSVD‐s vs. HC	ReHo‐GMV CSVD‐s < HC	Left caudate	44	5.295	−18	24	6
		Right caudate	42	5.090	15	24	12
	ReHo‐GMV CSVD‐s > HC	Right supplementary motor area	16	3.961	9	−15	51
	ALFF–GMV CSVD‐s < HC	Right caudate	173	5.295	15	27	0
		Left caudate	159	5.295	−18	27	6
	ALFF–GMV CSVD‐s > HC	Right inferior temporal gyrus	31	4.901	45	−21	−30
CSVD‐s vs. CSVD‐m	ReHo‐GMV CSVD‐s < CSVD‐m	Right caudate	56	5.167	18	27	−3
		Left caudate	61	4.957	−18	27	0
		Left insula	24	4.217	−36	−15	0
	ALFF–GMV CSVD‐s < CSVD‐m	Left caudate	181	5.295	−18	27	6
		Right caudate	137	5.295	15	21	15
		Right putamen	23	4.360	27	−9	3
	ALFF–GMV CSVD‐s > CSVD‐m	Left middle frontal gyrus	11	4.388	−36	60	6
		Left medial superior frontal gyrus	14	4.046	−6	51	48

*Note:* ANCOVA and LSD post hoc tests were used to identify the significantly altered coupling between groups after Gaussian random field (GRF) multiple comparison corrections (voxel level *p* < 0.001, cluster level *p* < 0.05).

Abbreviations: CSVD‐m, mild CSVD burden (score ≤ 1) group; CSVD‐s, severe CSVD burden (score ≥ 2) group; HC, healthy controls.

**FIGURE 2:**
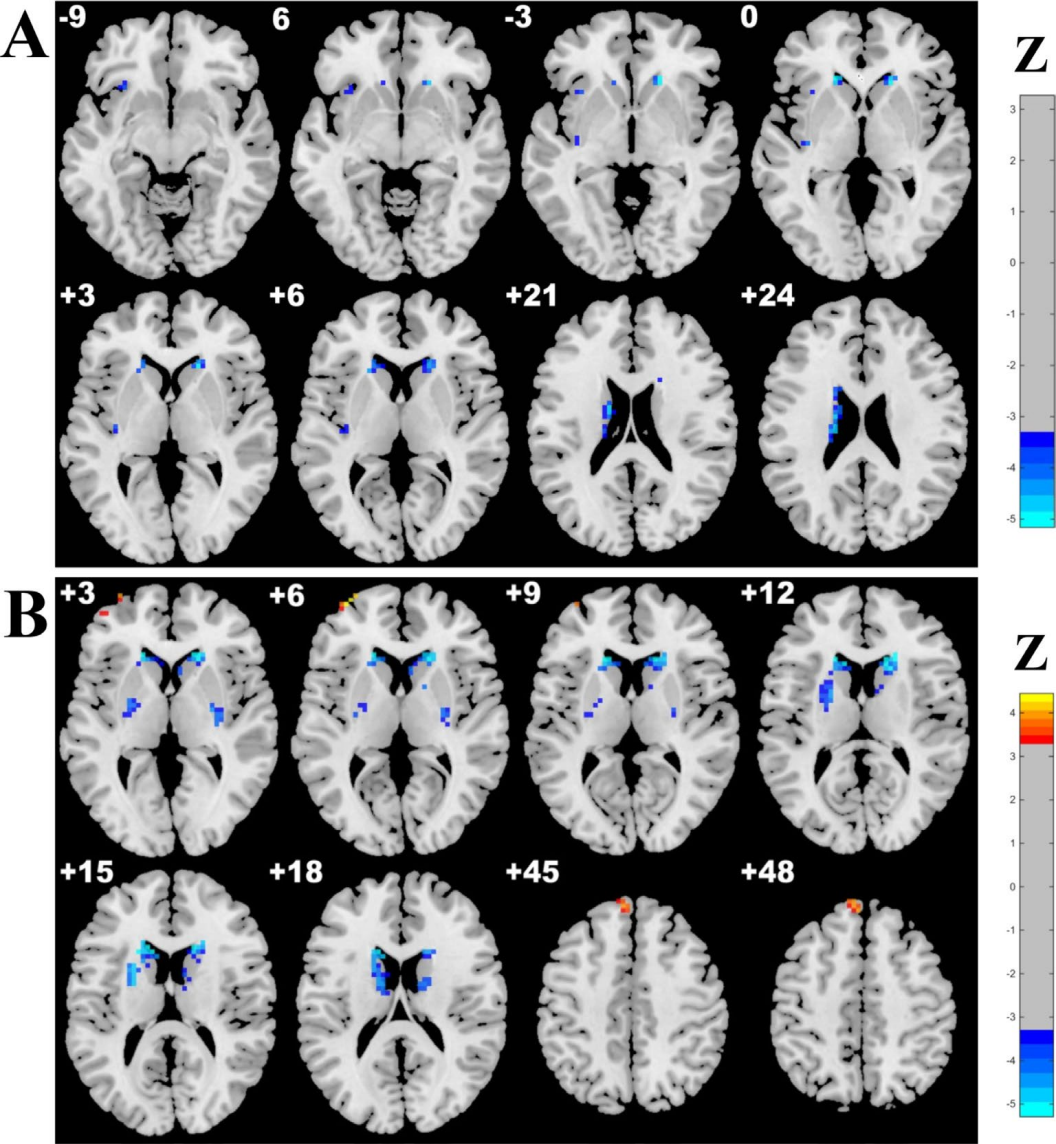
Brain clusters with significantly altered structure–function coupling in the CSVD‐s group relative to the CSVD‐m group. Significant differences in (A) ReHo–GMV and (B) ALFF–GMV coupling between CSVD‐s and CSVD‐m groups were identified using ANCOVA and LSD post hoc tests with GRF correction (voxel‐level *p* < 0.001, cluster‐level *p* < 0.05). Only clusters with significant differences in the CSVD‐s group are displayed. As shown, increased ALFF–GMV coupling was found in the left middle frontal gyrus (MFG) and medial superior frontal gyrus (SFGmed), whereas decreased ReHo–GMV and ALFF–GMV coupling were detected in the bilateral caudate nucleus, left insula, and right putamen. The blue‐light blue color bars represent decreased coupling, and red‐yellow represent increased coupling in the CSVD‐s group relative to CSVD‐m group.

Moreover, compared with the CSVD‐m group, the CSVD‐s group presented significantly decreased ReHo–GMV and ALFF–GMV coupling values in the bilateral CAU, decreased ReHo–GMV coupling value in the left insula, and ALFF–GMV coupling in the right putamen, and increased ALFF–GMV coupling value in the left medial superior frontal gyrus (SFGmed) and middle frontal gyrus (MFG). No significant difference in coupling was found between the CSVD‐m group and the HC group (Table 2 and Figure 2).

### 3.3 Mediation Analysis Results

**FIGURE 3:**
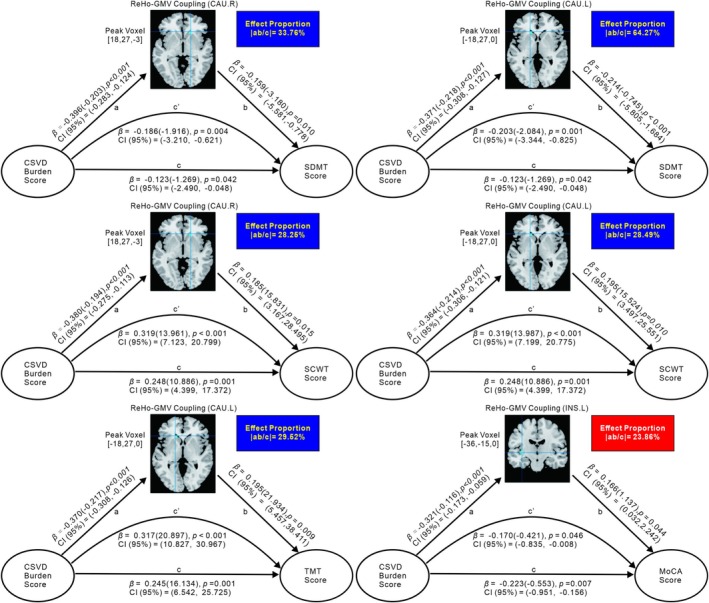
Simple mediation model for the correlation between CSVD burden and cognitive test score mediated by ReHo‐GMV coupling. Ab, indirect effect; c′, direct effect; *c*, total effect; *β*(): *β* = standardized regression coefficients, with unstandardized coefficients in bracket; CI (95%): 95% confidence intervals from 5000 bootstrapping iterations. The ReHo–GMV coupling in bilateral CAU, left CAU, and left insula were the mediators of CSVD burden score and SDMT/SCWT, TMT, and MoCA scores, respectively.

**FIGURE 4:**
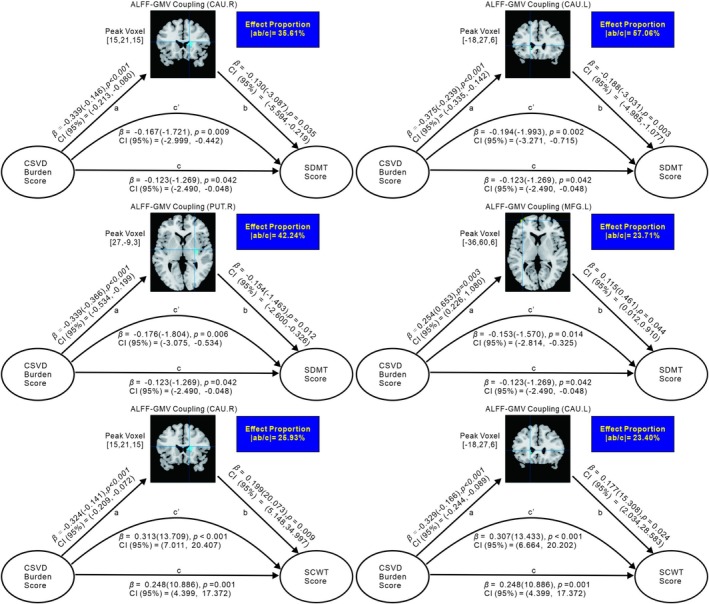
Simple mediation model for the correlation between CSVD burden and cognitive test score mediated by ALFF–GMV coupling. Ab, indirect effect; c′, direct effect; c, Total effect; *β*(): *β* = standardized regression coefficients, with unstandardized coefficients in bracket; CI (95%): 95% confidence intervals from 5000 bootstrapping iterations. The ALFF–GMV coupling in bilateral CAU, left MFG and right PUT were the mediators of CSVD burden score and SDMT score, and the ALFF–GMV coupling in bilateral CAU were the mediators of CSVD burden score and SCWT score.

The simple mediation model (Figures 3 and 4; Table S1) revealed that the presence of brain structure–function coupling played a significant mediating role in the relationship between CSVD burden and cognitive dysfunction after controlling for age, gender, education, and head motion as covariates. Specifically, we found that ReHo–GMV coupling significantly partially mediated the relationship between the CSVD burden score and the MoCA score in the left insula, where the effect proportion equaled the absolute value of the ratio of the indirect effect to the total effect.

Moreover, the correlations between the CSVD burden score and the SDMT/SCWT score were significantly mediated by ReHo–GMV and ALFF–GMV coupling in bilateral CAU. In addition, the relationship between the CSVD burden score and the SDMT score was significantly mediated by ALFF–GMV coupling in the left MFG and right putamen, whereas the relationship between the CSVD burden score and the TMT score was significantly mediated by ReHo–GMV coupling in the left CAU. Owing to the indirect and direct effects in the aforementioned mediating effects showing opposite signs, we considered them masking effects [37].

## 4 Discussion

To our knowledge, this is the first time to explore the mediating association among CSVD burden, brain structure–function coupling, and cognitive dysfunction. We found the following: (1) the CSVD patients had significantly decreased cognitive function with increasing CSVD burden; (2) the CSVD‐s group presented significantly altered voxel‐wise structure–function coupling (both ALFF–GMV and ReHo–GMV), mainly in the basal ganglia and frontotemporal lobe, compared with the control and CSVD‐m groups; (3) disrupted structure–function coupling in the bilateral CAU, left insula, and MFG, and right putamen was a mediator between CSVD burden and cognitive dysfunction. Our research suggests that cognitive dysfunction in patients with different CSVD burdens is affected by altered coupling in the basal ganglia and frontotemporal lobe.

On the basis of previous ALFF–GMV studies [15], we further supplemented the ReHo–GMV coupling, which reflects the structure–function coupling of a specific region or voxel [14]. In general, the decreased ALFF–GMV and ReHo–GMV coupling values were mainly found in the basal ganglia (including the bilateral CAU and right putamen) and left insula in CSVD patients. The reduced structure–function coupling of the basal ganglia may be related to gait disorders and the decline in language ability, memory, and overall cognition in CSVD patients [15]. The nsula is an important structure connecting the basal ganglia and the limbic system and is crucial for maintaining normal cognitive function. Evidence has shown that insula impairment is associated with cognitive impairment in all neuropsychological fields [38]. In contrast, increased coupling values were found in the frontotemporal lobe (including the right ITG and SMA, left MFG and SFGmed). We noted in previous studies that CSVD‐induced cognitive impairment may be related to inferior temporal cortical thinning and frontoparietal hypometabolism [15]. Moreover, the SMA is a significant part of the sensorimotor network, mediating action initiation and participating in motor processes [39]. Executive function and cognitive control can be affected due to the damage of the SMA region [40]. Scientists have demonstrated structural or functional changes in the SMA [41, 42] and insula [38] in patients with CSVD, which supported our findings.

Previous studies have confirmed that brain structural or functional neuroimaging indicators may be important mediators of age‐ or disease‐related cognitive changes [16, 17]. We found that ALFF–GMV coupling only mediated the relationship between the CSVD burden and the SD/SCWT, whereas ReHo–GMV coupling mediated the relationship between the CSVD burden and the TMT/MoCA in addition to the SDMT/SCWT. Thus, ReHo–GMV coupling is also a pregnant imaging indicator used to explore the association between CSVD burden and cognitive dysfunction [14]. Interestingly, with respect to the type of mediating effect, except for the left insula, we observed that the ReHo–GMV/ALFF–GMV coupling values of various brain regions played masking roles in the association between CSVD burden and cognitive impairment. The masking effect refers to the phenomenon where the total effect between the independent and dependent variables is either nonsignificant or in the opposite direction when the mediator is not included; however, after introducing the mediator, the direct effect becomes significantly stronger and reverses direction. This indicates that the mediator partially “masks” the true relationship between the independent and dependent variables. In our study, the indirect effect through structure–function coupling and the direct effect of CSVD burden on cognition had opposite signs, indicating that changes in ReHo–GMV/ALFF–GMV coupling values in these brain regions enhance the effect of CSVD burden on SDMT/SCWT/TMT‐related cognitive dysfunction. This suggests that coupling abnormalities in these regions may aggravate, rather than mitigate, the cognitive consequences of CSVD. Such a pattern may reflect impaired compensatory responses or maladaptive reorganization, highlighting the pathological role of disrupted coupling in exacerbating cognitive decline.

An increase in the CSVD burden can lead to damage to brain structure and function and cognitive decline [42], which is related to the increased severity of pathological changes. With the progression of CSVD, inflammation of the central nervous system gradually leads to neuronal dysfunction, apoptosis, and necrosis [43]. Therefore, patients in the CSVD‐s group had more severe brain damage, and it is not difficult to understand the more significant decoupling in this group. Notably, both the ALFF–GMV and ReHo–GMV coupling values of the bilateral CAU significantly mediated the CSVD burden and cognitive functions (SDMT/SCWT) in our research. Considering that the ALFF–GMV and ReHo–GMV coupling values of the bilateral CAU were significantly reduced in CSVD patients, we can infer that the CAU is a key region affected by CSVD and is closely related to the cognitive impairment caused by CSVD. Previous studies have shown that the CAU participates in executive functions, such as working memory, cognitive control, and attention [44], which is consistent with the detection range of the SDMT and SCWT. Moreover, the cognitive impairments caused by CSVD include mainly executive function and cognitive processing speed [45].

In our study, the right putamen and left MFG were also important brain regions that exerted masking effects. On the one hand, the putamen and CAU together make up the neostriatum, which is a crucial component of the basal ganglia. Scientists have previously proposed “remaining cognitive functions” of the basal ganglia, involving association, memory and complex perceptual processes [46]. On the other hand, the MFG belongs to the dorsal prefrontal cortex, which is involved in executive functions and higher cognitive processes [47]. The effects of vascular lesions, including CSVD [48], on cognitive impairment are mediated in part by altered functional connectivity, especially in the frontal lobe [49]. Moreover, the basal ganglia and frontal cortex are located in the cortico–basal ganglia circuitry, working together to develop and execute complex behaviors [47]. Because the clustering and network efficiency of CSVD are significantly decreased, the information transmission between regions is reduced [8]. Therefore, when the burden of CSVD increases, it may disrupt structure–function coupling and connectivity in these brain regions, enhancing the effect of CSVD on cognitive impairment.

Intriguingly, we found that only the ReHo–GMV coupling value of the left insula partially mediated the association between CSVD burden and MoCA‐related cognitive impairment; that is, it attenuated the association between them. We speculated that this may be due to the specific role of the insula in the brain. Burzynska et al. [50] identified the insula as a “connector hub” in the structural and functional brain network, which, together with the lateral occipital, parietal, dorsal superior frontal cortex, and premotor regions, forms the “rich club” regions (structures within this region exhibit a high degree of interconnectivity). The decrease in ReHo–GMV coupling in the insula may initiate a compensatory increase in “rich‐club” region coupling, thereby attenuating the cognitive dysfunction caused by CSVD.

There were several limitations. First, the cross‐sectional design precludes causal inference, and the relatively small sample size of the CSVD‐s group may reduce statistical power, particularly for mediation and subgroup analyses involving multiple brain regions and cognitive domains. Future longitudinal studies with larger and more balanced samples are needed to clarify the temporal dynamics among CSVD burden, coupling alterations, and cognitive decline. Second, although structural and functional data were rigorously processed, histopathological validation was not possible due to the noninvasive nature of MRI. Third, our analysis focused on voxel‐wise coupling within individual brain regions, without examining inter‐regional connectivity, which may also contribute to cognitive dysfunction. Fourth, potential confounding factors—such as vascular risk factors (e.g., hypertension and diabetes) and lifestyle behaviors (e.g., smoking, alcohol use, and physical activity)—were not fully accounted for. These variables may impact brain structure and function, and should be incorporated into future studies to strengthen the validity of the findings. Finally, although the group‐level differences in voxel‐wise coupling parameters were corrected for multiple comparisons using Gaussian Random Field (GRF) theory, no additional multiple comparison correction was applied to mediation analyses. This aligns with established Bootstrap methodology [35, 36], where significance is determined via CIs rather than *p*‐value adjustments. However, we acknowledge that the mediation results should be interpreted with caution due to the risk of false positives from multiple testing, and future studies with larger samples should prioritize hypothesis‐driven confirmatory analyses to validate these findings.

In conclusion, brain structure–function coupling characterized by ReHo–GMV and ALFF–GMV in the basal ganglia and frontotemporal lobe indeed mediates the cognitive dysfunction caused by CSVD and is an innovative and effective neuroimaging indicator to further elucidate the complex relevancy between CSVD burden and cognitive dysfunction. Our study provides a novel insight into the neurophysiological mechanism of CSVD and is momentous for targeted therapy for this disease.

## Author Contributions

N.W., H.W., C.L., and L.G. were involved in the conception, design, and conduct of the study and the analysis and interpretation of the results. N.W. wrote the first draft of the manuscript, and all the authors were involved in data collection. L.G. is the guarantor of this work and, as such, had full access to all the data in the study and takes responsibility for the integrity of the data and the accuracy of the data analysis.

## Conflicts of Interest

The authors declare no conflicts of interest.

## Supporting information


**Appendix S1:** cns70604‐sup‐0001‐AppendixS1.docx.

## Data Availability

The datasets generated or analyzed during the study are available from the corresponding author L.G. upon reasonable request.
