# Supplementary material for: Disrupted Brain Structure–Function Coupling and Its Mediating Effects on the Associations Between Cerebral Small Vessel Disease Burden and Cognitive Dysfunction
Source: CNS Neurosci Ther. 2025 Sep 18;31(9):e70604. doi: 10.1111/cns.70604 (PMC12445204; doi:10.1111/cns.70604)
Supplement: Supplementary file 1 — Appendix S1: cns70604‐sup‐0001‐AppendixS1.docx. [file CNS-31-e70604-s001.docx]

**Data preprocessing**

The main preprocessing steps for fMRI and T1W images were performed via the Data Processing & Analysis for Brain Imaging toolbox (DPABI v6.0, <http://rfmri.org/dpabi>) as follows: (1) The first 10 volumes of each functional scan were removed. (2) Slice timing and head motion correction were performed, and subjects were excluded if their maximum rotation was >3.0°, maximum translational displacement was >3.0 mm, or mean framewise displacement (FD) was >0.2 mm ^1^. (3) The T1W images were co-registered to the mean functional images. (4) The T1W images were segmented into GM, WM, and cerebrospinal fluid (CSF) via the new segment tool. (5) The segmented GM images were normalized to the Montreal Neurological Institute (MNI) standard space to obtain deformation information via the DARTEL algorithm ^2^. Normalized GM images were then modulated to ensure that the actual GMV was well preserved following spatial normalization. The modulated GMV maps were resampled to a voxel size of 3×3×3 mm^3^ and then smoothed with an isotropic Gaussian kernel of 4 mm full width at half maximum (FWHM) for subsequent coupling analysis. (6) Nuisance signals, including CSF, WM and Friston-24 head motion parameters ^3^, were regressed out for functional images. (7) The functional images were normalized to the MNI space by deformation fields derived from corresponding GM images and resampled to 3×3×3 mm^3^ voxel resolution. (8) The functional image was spatially smoothed with an isotropic Gaussian kernel of 4 mm full width at half maximum (FWHM). (9) For smoothed functional images, the voxel-wise signal was converted from the time domain to the frequency domain via fast Fourier transform. The ALFF in a given voxel is calculated as the average square root value of the power spectrum in the frequency range 0.01–0.08 Hz ^4^. The ALFF per voxel was subsequently divided by the global mean ALFF to generate the standardized ALFF metric for subsequent coupling analysis. (10) The ReHo map was calculated before spatial smoothing of the functional image. After bandpass filtering (0.01–0.08 Hz) was performed on the normalized functional images to reduce the effects of low-frequency drift and high-frequency physiological noise, a ReHo map was generated by calculating Kendall’s coefficient of concordance (KCC) of the time series for a given voxel with those of its nearest 26 neighbors ^5^. Finally, the ReHo per voxel was divided by the global mean ReHo to generate the standardized ReHo metric for further coupling analysis.

**References**

1. Jenkinson M, Bannister P, Brady M, Smith S. Improved optimization for the robust and accurate linear registration and motion correction of brain images. *Neuroimage.* 2002;17(2):825-841.

2. Ashburner J. A fast diffeomorphic image registration algorithm. *Neuroimage.* 2007;38(1):95-113.

3. Friston KJ, Williams S, Howard R, Frackowiak RSJ, Turner R. Movement‐Related effects in fMRI time‐series. *Magnetic Resonance in Medicine.* 1996;35(3):346-355.

4. Yu-Feng Z, Yong H, Chao-Zhe Z, et al. Altered baseline brain activity in children with ADHD revealed by resting-state functional MRI. *Brain and Development.* 2007;29(2):83-91.

5. Zang Y, Jiang T, Lu Y, He Y, Tian L. Regional homogeneity approach to fMRI data analysis. *Neuroimage.* 2004;22(1):394-400.

**Table S1.** Mediating effects of brain structure-function coupling on relationships of CSVD burden score with cognitive score.

| **Mediator** | **Effect**  **Type** | **Dependent variable** | **Effects** | **Effect Value** | **Boot SE** | **Boot LLCI** | **Boot ULCI** | **Effect Ratio** |
| --- | --- | --- | --- | --- | --- | --- | --- | --- |
| ReHo-GMV coupling of CAU.R | Masking effect | SDMT | Indirect effects | 0.647 | 0.281 | 0.161 | 1.241 | 33.76% |
|  |  |  | Direct effect | -1.916 | 0.655 | -3.210 | -0.621 | -- |
|  |  |  | Total effect | -1.269 | 0.618 | -2.490 | -0.048 | -- |
|  |  |  |  |  |  |  |  |  |
| ReHo-GMV coupling of CAU.L | Masking effect | SDMT | Indirect effects | 0.815 | 0.325 | 0.303 | 1.576 | 64.27% |
|  |  |  | Direct effect | -2.084 | 0.638 | -3.344 | -0.825 | -- |
|  |  |  | Total effect | -1.269 | 0.618 | -2.490 | -0.048 | -- |
|  |  |  |  |  |  |  |  |  |
| ReHo-GMV coupling of CAU.R | Masking effect | SCWT | Indirect effects | -3.075 | 1.360 | -5.887 | -0.658 | 28.25% |
|  |  |  | Direct effect | 13.961 | 3.461 | 7.123 | 20.799 | -- |
|  |  |  | Total effect | 10.886 | 3.283 | 4.399 | 17.372 | -- |
|  |  |  |  |  |  |  |  |  |
| ReHo-GMV coupling of CAU.L | Masking effect | SCWT | Indirect effects | -3.101 | 1.314 | -6.128 | -0.895 | 28.49% |
|  |  |  | Direct effect | 13.987 | 3.435 | 7.199 | 20.775 | -- |
|  |  |  | Total effect | 10.886 | 3.283 | 4.399 | 17.372 | 10.886 |
|  |  |  |  |  |  |  |  |  |
| ReHo-GMV coupling of INS.L | Partial  mediation | MoCA | Indirect effects | -0.132 | 0.065 | -0.273 | -0.021 | 23.86% |
|  |  |  | Direct effect | -0.421 | 0.209 | -0.835 | -0.008 | -- |
|  |  |  | Total effect | -0.553 | 0.201 | -0.951 | -0.156 | -- |
|  |  |  |  |  |  |  |  |  |
| ReHo-GMV coupling of CAU.L | Masking effect | TMT | Indirect effects | -4.763 | 2.866 | -11.144 | -0.044 | 29.52% |
|  |  |  | Direct effect | 20.897 | 5.097 | 10.827 | 30.967 | -- |
|  |  |  | Total effect | 16.134 | 4.855 | 6.542 | 25.725 | -- |
|  |  |  |  |  |  |  |  |  |
| ALFF-GMV coupling of CAU.R | Masking effect | SDMT | Indirect effects | 0.452 | 0.222 | 0.068 | 0.919 | 35.61% |
|  |  |  | Direct effect | -1.721 | 0.647 | -2.999 | -0.442 | -- |
|  |  |  | Total effect | -1.269 | 0.618 | -2.490 | -0.048 | -- |
|  |  |  |  |  |  |  |  |  |
| ALFF-GMV coupling of CAU.L | Masking effect | SDMT | Indirect effects | 0.724 | 0.257 | 0.274 | 1.287 | 57.06% |
|  |  |  | Direct effect | -1.993 | 0.647 | -3.271 | -0.715 | -- |
|  |  |  | Total effect | -1.269 | 0.618 | -2.490 | -0.048 | -- |
|  |  |  |  |  |  |  |  |  |
| ALFF-GMV coupling of PUT.R | Masking effect | SDMT | Indirect effects | 0.535 | 0.210 | 0.164 | 0.976 | 42.24% |
|  |  |  | Direct effect | -1.804 | 0.643 | -3.075 | -0.534 | -- |
|  |  |  | Total effect | -1.269 | 0.618 | -2.490 | -0.048 | -- |
|  |  |  |  |  |  |  |  |  |

| ALFF-GMV coupling of MFG.L | Masking effect | SDMT | Indirect effects | 0.301 | 0.161 | 0.022 | 0.652 | 23.71% |
| --- | --- | --- | --- | --- | --- | --- | --- | --- |
|  |  |  | Direct effect | -1.570 | 0.630 | -2.814 | -0.325 | -- |
|  |  |  | Total effect | -1.269 | 0.618 | -2.490 | -0.048 | -- |
|  |  |  |  |  |  |  |  |  |
| ALFF-GMV coupling of CAU.R | Masking effect | SCWT | Indirect effects | -2.823 | 1.228 | -5.337 | -0.625 | 25.93% |
|  |  |  | Direct effect | 13.709 | 3.390 | 7.011 | 24.407 | -- |
|  |  |  | Total effect | 10.886 | 3.283 | 4.399 | 17.372 | -- |
|  |  |  |  |  |  |  |  |  |
| ALFF-GMV coupling of CAU.L | Masking effect | SCWT | Indirect effects | -2.547 | 1.177 | -5.096 | -0.564 | 23.40% |
|  |  |  | Direct effect | 13.433 | 3.426 | 6.664 | 20.202 | -- |
|  |  |  | Total effect | 10.886 | 3.283 | 4.399 | 17.372 | -- |

Note: MoCA: Montreal cognitive assessment; SDMT: symbol digit modalities test; SCWT: sum of Stroop color-word test (stroop1-3); TMT: the trail-making test. MFG.L: left middle frontal gyrus; INS.L: left insula; CAU.L/R: left/right caudate; PUT.R: right putamen; Boot SE, bootstrap standard error; Boot LLCI, bootstrap lower limit 95% confidence interval; Boot ULCI, bootstrap upper limit 95% confidence interval. Effect ratio: the absolute value of the ratio of the indirect effect to the total effect.
